# Multiscale nest‐site selection of ducks in the western boreal forest of Alberta

**DOI:** 10.1002/ece3.9139

**Published:** 2022-07-31

**Authors:** Matthew E. Dyson, Stuart M. Slattery, Bradley C. Fedy

**Affiliations:** ^1^ School of Environment, Resources and Sustainability University of Waterloo Waterloo Ontario Canada; ^2^ Institute for Wetlands and Waterfowl Research Ducks Unlimited Canada Stonewall Manitoba Canada

**Keywords:** industrial development, linear features, nesting ecology, oil and gas, spatial prediction, third‐order, waterfowl

## Abstract

There is limited data regarding the nesting ecology of boreal ducks and their response to industrial development, despite this region being an important North American breeding area. We investigated how landcover and oil and gas development affect third‐order nest‐site selection of boreal ducks. We located duck nests in Alberta's western boreal forest between 2016 and 2018. We used multiscale analysis to identify how scale affects the selection of a resource using generalized linear mixed‐effects models and determined what scale‐optimized combination of landscape features were most important in describing where ducks nest. We located 136 nests of six species of upland nesting ducks between 2016 and 2018. The magnitude, direction, and best spatial scale varied by resource. For landcover, ducks selected nest‐sites associated with mineral wetlands (300 m) and open water (300 m). Ducks avoided greater densities of seismic lines (300 m) and pipelines (2500 m) but selected nest‐sites associated with borrow pits (300 m) and roads (1000 m). We used our models to predict important duck nesting habitat in the boreal forest, which can support conservation and management decisions. We recommend conservation actions target the conservation of mineral wetlands and associated habitats within this working landscape. Further research is necessary to understand the adaptive consequences of nest‐site selection and how industrial development influences important nest predators.

## Graphical abstract

There is limited data regarding the nesting ecology of boreal ducks and their response to industrial development, despite this region being an important North American breeding area. We investigated how landcover and oil and gas development affect third‐order nest‐site selection of boreal ducks. We developed multiscale resource functions to predict critical nesting habitat for ducks, which provides a decision‐support tool for conservation managers in this working landscape.

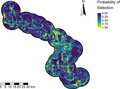

## 1 INTRODUCTION

Investigation of how anthropogenic land use alters resource selection patterns by animals is of imminent conservation relevance (Allred et al., 2015; Fahrig, 2003; Muhly et al., 2019). In the boreal forest, rapid growth of oil and gas development in recent decades has created habitat loss and fragmentation because of industrial block features (e.g., well pads and industrial facilities) and linear features (e.g., roads, pipelines, and seismic lines), respectively (Carlson & Browne, 2015; Fisher & Burton, 2018; Hebblewhite, 2017; Pickell et al., 2015; Schneider & Dyer, 2006). These working landscapes (Stewart et al., 2019) are composed of a heterogeneous mosaic of natural and anthropogenic features resulting in altered species space use patterns and interactions that have benefitted some species (e.g., generalists) to the detriment of others (e.g., specialists) (Fisher & Burton, 2018).

Mammalian predators, such as wolves (*Canis lupus*) and American black bears (*Ursus americanus*), have generally benefitted from industrial development as a result of increased access to prey facilitated by linear features (DeMars & Boutin, 2018; Dickie et al., 2017; Ehlers et al., 2014; Tigner et al., 2014). Conversely, prey species, such as woodland caribou (*Rangifer tarandus caribou*), have declined, in part, because fragmentation of refugia has increased spatial overlap with predators (DeMars & Boutin, 2018; Ehlers et al., 2016; Mumma et al., 2017, 2018). For birds, species associated with older intact forests have declined in association with industrial development, while species associated with more open forests have increased (Bayne et al., 2016; Mahon et al., 2019). Quantifying species responses and associations is complicated by mounting evidence that species responses to habitat and industrial development vary across scales (Bayne et al., 2016; Decesare et al., 2012; Mumma et al., 2019; Stewart et al., 2019; Toews et al., 2017). For example, wolves, caribou, and moose (*Alces alces*), exhibited scale‐dependent resource selection with varying availability in northeastern British Columbia, where wolf selection for seismic lines tended to increase with expanding available scales (Mumma et al., 2019). Managers must decide the appropriate spatial extents for the implementation of management actions. Therefore, it is critical to assess the scale dependence of species resource use patterns in response to industrial disturbance to make effective management recommendations that aim to reduce the impacts of disturbance.

Scale‐dependent selection of a resource by animals is often conceptualized through hierarchical orders of selection (Decesare et al., 2012; Johnson, 1980; Meyer & Thuiller, 2006). However, within and across orders of selection, animal responses occur along a scale continuum and the best scale of response is often species and resource specific (Boyce et al., 2017; Martin & Fahrig, 2012; Mayor et al., 2007, 2009; Meyer & Thuiller, 2006). Termed the functional response (Mysterud & Ims, 1998), selection for a given resource will be conditional on its availability (Northrup et al., 2013). Multiscale resource selection functions (MRSFs) provide an explicit framework to incorporate and understand an animal's functional response to resources across spatial scales (Bauder et al., 2018; Laforge, Brook, et al., 2015; Laforge, Vander Wal, et al., 2015; McGarigal et al., 2016).

Ducks (Family: Anatidae) are migratory, crossing large landscapes each breeding season, and must make annual settling decisions that represent selection across spatial scales. Most ducks in North America settle in the prairie pothole region but the boreal forest is the second most important breeding area supporting up to 41% of North American waterfowl populations annually (Barker et al., 2014; Slattery et al., 2011). The decision of where to nest is one of the most important choices a duck makes and nest success plays an important role in regulating duck populations and is influenced by predation and environmental conditions (Coluccy et al., 2008; Hoekman et al., 2002; Howerter et al., 2014; Koons et al., 2014). Additionally, nest site selection influences the probability of hen survival, access to forage during incubation, and options for brood‐rearing habitat (Dyson et al., 2018; Gibson et al., 2016). Therefore, patterns of nest‐site selection should represent long‐term optima and provide insight into identifying important nesting habitats (Clark & Shutler, 1999).

Where ducks choose to nest relative to habitats available within their breeding home range (i.e., third‐order selection; sensu Johnson, 1980) is likely the most relevant for informing conservation decisions (Howerter et al., 2008; Smith et al., 2020; Stephens et al., 2005). Within the third order of selection, investigating multiscale responses of nest‐site selection of boreal ducks provides an opportunity to integrate scientific knowledge with management decisions (Roberts et al., 2017). Our objective was to evaluate how industrial development and habitat influence nest‐site selection of upland nesting ducks in the boreal forest. We used a multiscale analysis to identify scale‐dependent resource selection and identified what scale‐optimized combination of landscape features were most important in describing where ducks nest at the third order of selection (McGarigal et al., 2016). We spatially predicted our models to identify important duck nesting habitat in the boreal forest with the goal of helping inform and advance the conservation and management of boreal ducks.

## 2 METHODS

### 2.1 Field sampling

**FIGURE 1:**
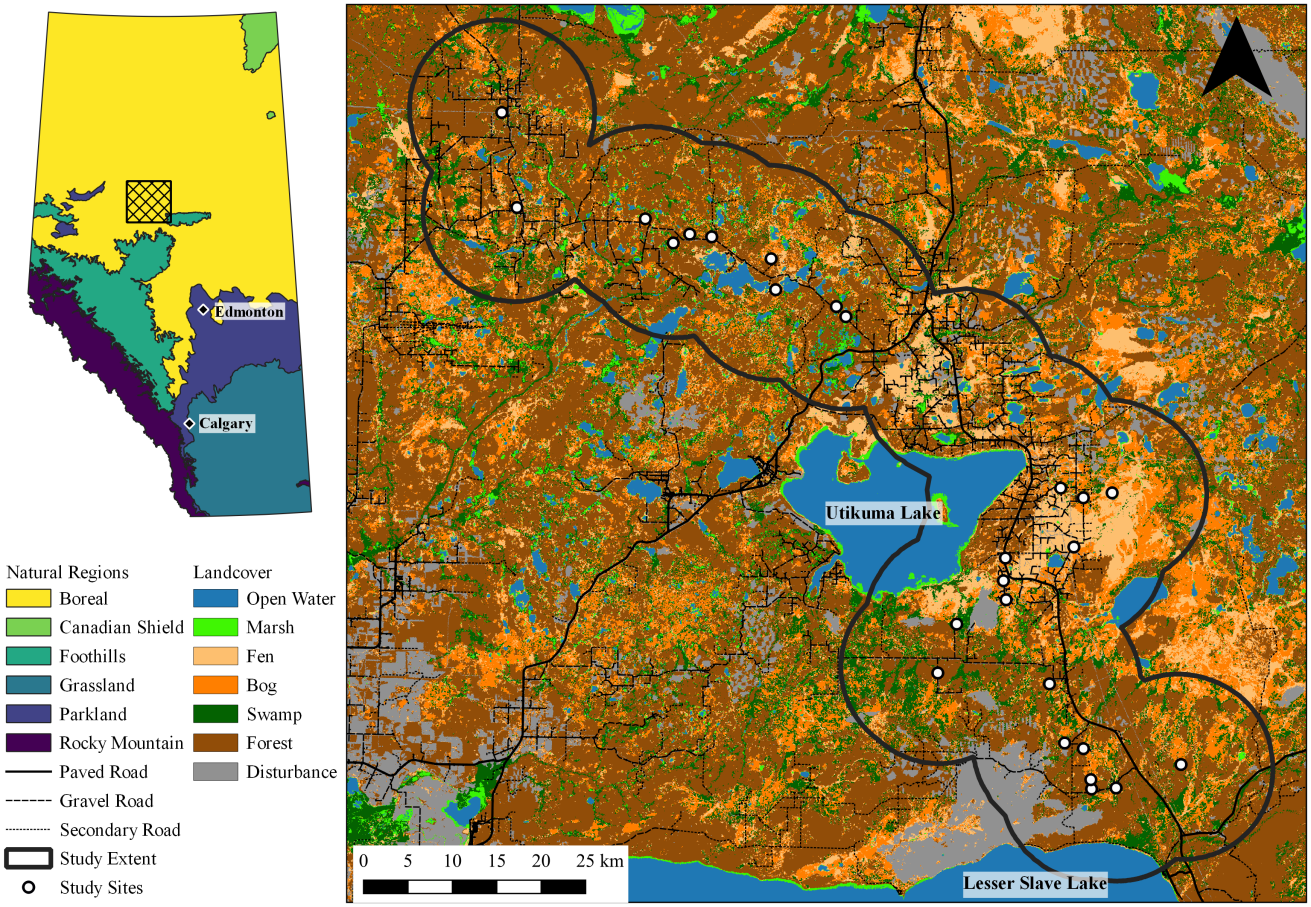
Map of study extent and nest searching site locations (*n* = 26) from 2016 to 2018 with the enhanced wetland classification (EWC) landcover layer. The inlay highlights the location of the study area in the boreal forest of Alberta, Canada in the context of the provincial natural regions.

Our study area was located north of Slave Lake, Alberta, Canada, near Utikuma Lake in Canada's Boreal Plains ecozone and Alberta's boreal forest natural region, hereafter, the western boreal forest (WBF; Figure 1). The landscape is a mosaic of deciduous, mixed wood, and coniferous forests interspersed by extensive wetland complexes and industrial development. Historically, land cover has been shaped by natural disturbance events, such as insect outbreaks and wildfire (Carlson & Browne, 2015). Over the previous decades, landcover has been dramatically changed by historical natural drivers (i.e., forest fires) and increasing activity from industrial development related to forestry and oil and gas exploration and extraction (Carlson et al., 2015; Dawe et al., 2014). Industrial development has resulted in the creation of high density linear features (e.g., seismic lines, roads, and pipelines) and large block features (e.g., well pads, pumping stations, and industrial sites) that did not previously exist on the landscape (Fisher & Burton, 2018; Schneider & Dyer, 2006).

We selected sites for nest‐searching using hierarchical selection criteria using spatial data obtained from Ducks Unlimited Canada (DUC) and the Alberta Biodiversity Monitoring Institute (ABMI; ABMI, 2017). We used a 2.5 × 2.5 km sampling grid that was used in a companion study for aerial surveys (Ducks Unlimited Canada, 2014). Generally, nests of upland nesting duck species are in proximity to wetland habitats that provide interspersed vegetation for brood rearing (Baldassarre, 2014; Batt et al., 1992). We had no previous experience with nest searching on this landscape and expected nest density to be low compared to more commonly studied regions, such as Arctic or Prairies given lower pair settling densities. Therefore, to maximize our probability of locating nests, we used predictive models of duck pair density (Barker et al., 2014). We only considered study grids with a predicted duck density greater than the median for the region in our candidate set of grids targeted for nest searching (4 pairs/2.5 km^2^; Barker et al., 2014). Next, we excluded grids that were recently logged or burned (within 20 years) because we were interested in understanding the effects of oil and gas development independent of other disturbances. Next, we used a cumulative industrial development footprint (i.e., area of wells, roads, pipelines, seismic lines, and industrial features) to categorize study grids across a gradient of industrial development divided into three categories: high, medium, and low. Using the remaining candidate grids, we selected one unique wetland study sites from within a grid for nest searching. A final selection criterion required sites to be accessible (i.e., within ~3 km of a vehicle accessible road) and contain at least one water body (>1 ha) for logistical purposes. Therefore, our selected sites covered the gradient of industrial development present in the region and provided good spatial coverage across our study extent.

We searched 16 sites in 2016, 24 sites in 2017, and 25 sites in 2018 between May 1 and July 31 (Figure 1). We searched most sites in subsequent years, except for one site that was searched only in 2016 and one site that was searched in 2016 and 2018. We completed two searches of each site in 2016, two to three searches of each site in 2017, and three searches of each site in 2018. Search efforts were separated by 15–25 days and were performed on foot by teams of three to six searchers. Teams systematically searched around wetlands by disturbing vegetation to increase the probability of detecting an incubating female (Klett et al., 1986). Our target species were any upland nesting duck. We estimated searched area size for each site by buffering GPS search tracks from all searchers and years by 20 m, dissolving the buffers together, and calculating an area, which resulted in sites with a mean searched area of 27.46 ± 12.15 ha ($\bar{x}$ ± SD). We searched for nests between 0800 h and 1600 h (Gloutney et al., 1993) and located an additional three radio‐tagged mallard nests in 2018 with VHF telemetry as part of a pilot study. Nests of marked birds were found in similar habitat to unmarked birds, so we combined them with our sample. In addition, nests found incidentally during monitoring or other associated field work were included in our sample. When a nest was located, we identified the duck species, recorded the number of eggs (i.e., clutch size), and estimated the nest age by candling and floating eggs (Dyson et al., 2019; Klett et al., 1986; Weller, 1956).

### 2.2 Landscape predictors

We developed a suite of landscape predictors based on spatial layers that represented land cover and land use features, which we predicted to be important for duck nest‐site selection. We provide summaries of these variables and their distributions for nests and available sites and for our searched areas and within the study extent (Table 1). Land cover variables were developed from Ducks Unlimited Canada's Enhanced Wetland Classification layer at a 30‐m resolution (Ducks Unlimited Canada, 2011) and were summarized in four thematic groups: mineral wetlands, peatlands, open water, and forest. Mineral wetlands included swamp, emergent marsh, aquatic beds, mudflats, and meadow marsh; peatlands included bogs and fens; open water included open water; and forest included conifer, deciduous, and mixedwood forests (Ducks Unlimited Canada, 2011).

**TABLE 1:** Summary of variables used in the development of macrohabitat multiscale nest‐site selection models including their summary statistics of the mean and range for the study extent, estimated searched area, used, and available locations. Summary statistics were calculated from 30 m moving window surfaces.

Name	Units	Study extent	Searched	Used	Available
Mineral wetland	%	0.14 (0–1)	0.21 (0–0.61)	0.23 (0–1)	0.14 (0–1)
Peatland	%	0.10 (0–1)	0.37 (0–0.89)	0.34 (0–1)	0.29 (0–1)
Open water	%	0.08 (0–1)	0.04 (0–0.19)	0.09 (0–1)	0.06 (0–1)
Forest	%	0.46 (0–1)	0.33 (0–0.72)	0.25 (0–1)	0.46 (0–1)
Borrow pits	m^2^	8.18 (0–4500)	71.17 (0–4500)	351.6 (0–4287.2)	13.64 (0–4500)
Wells	m^2^	27.91 (0–8740.34)	74.21 (0–4500)	18.57 (0–2488.99)	40.24 (0–4281.81)
Pipelines	m	3.37 (0–510.03)	5.38 (0–318.27)	5.96 (0–180.89)	5.26 (0–225.62)
Roads	m	3.99 (0–675.47)	16.2 (0–320.5)	16.07 (0–180.03)	5.73 (0–223.03)
Seismic	m	29.59 (0–750.22)	21.95 (0–472.6)	14.73 (0–199.52)	33.87 (0–387.26)

Land use layers were developed from the Alberta Biodiversity Monitoring Institute's (ABMI) Human Features Inventory database (ABMI, 2017). These layers were retrieved as vectors which we converted to rasters (i.e., pixels) to facilitate analysis. We converted polygonal features to rasters by calculating the percent area (i.e., percent cover) within a pixel and line features were converted by calculating the sum of the length of each line feature in a pixel (Table 1). We quantified borrow pits, which included all borrow pits, sumps, dugouts, and lagoons; pipelines, which included any under or overground pipes of substantial length and capacity used for conveyance of petrochemicals; roads included all paved, gravel, vegetated, and winter roads and trails; seismic lines included all seismic line feature types; and wells included all active and inactive wells (ABMI, 2017). We excluded some features from analysis because they were sparse on the landscape (e.g., transmission lines and industrial buildings).

All land cover and land use variables were mapped at a 30‐m resolution across our study area. Therefore, we used a constant minimum 30 m resolution in our multiscale analysis described below (Timm et al., 2016). We summarized the landscape predictors at multiple spatial scales using moving windows (Hagen‐Zanker, 2016). We selected moving window radii sizes based on duck biology and management relevance (Ducks Unlimited Canada, 2014; Wheatley & Johnson, 2009). Specifically, we investigated moving window radii sizes of (30, 90, 300, 1000, 2500, and 5000 m). We considered moving windows less than 1000 m to be fine scale and consistent with expected movement of a breeding hen within her nesting home range (Cowardin et al., 1995; Ducks Unlimited Canada, 2014; Howerter, 2003). We considered moving windows greater than 1000 m coarse scale and expected resources selected at this scale to be more consistent with predator ecology (Fisher & Burton, 2018; Tigner et al., 2014). Moving window analysis calculated the mean resource value within the moving window radius for each pixel except for linear features, which were summarized as the total length of resource within each window for each pixel.

### 2.3 Study extent and used and available points

We defined the spatial extent of our study by generating a 10‐km buffer around all nest locations and the centroids of searched areas. Prior to generating pseudo‐absence points, we generated an exponential decay surface of the form *e* ^*(−d/α)*^ where *d* represents road distances from each pixel centroid and *α* was fixed to 3 km (Fedy et al., 2014), because we selected sites to search that were within 3 km of a road. The resulting layer was a probability surface, where pixels within 3 km of roads had a probability close to 1 and pixels outside of 3 km of roads had probabilities approaching 0. We used the decay surface to generate pseudo‐absence locations so that our availability sample was more consistent with our nest searching efforts (van Wilgenburg et al., 2015). We generated pseudo‐absence locations at a ratio of 20:1 to ensure we saturated the landscape with available locations to accurately quantify the heterogeneity on the landscape (Fedy et al., 2014; Northrup et al., 2013).

### 2.4 Statistical analysis

We extracted data for each nest and non‐nest location for all landscape predictors and respective moving window sizes. Next, we standardized ($\bar{x}$ − *x*/SD) all data to improve computation, model fit, and prediction. We then developed multiscale resource selection functions (MRSFs) (Laforge, Brook, et al., 2015; Laforge, Vander Wal, et al., 2015). We used a weighted generalized linear mixed‐effects model with species as a random effect and a logit link as the base form of our MRSF, where the weighting was used to account for the skewed nest to non‐nest ratio (1:20), so that nest and non‐nest locations contributed equally in the model (Muff et al., 2020). We fit models using the lme4 package in R (Bates et al., 2016; R Core Team, 2020).

We used a pseudo‐optimized multiscale approach to identify the best scale for each landscape predictor in a univariate modeling framework from our six spatial scales and used AICc to select the best scale (Bauder et al., 2018; McGarigal et al., 2016). We then combined all covariates at their best pseudo‐optimized spatial scale into a multiscale global model and tested for collinearity between the covariates using a Pearson's r > |0.65| as the cutoff (Dormann et al., 2013). When we identified correlated variables, we allowed the individual variables to remain in the model set, but did not allow them to occur in the same model. We then tested all combinations of the fully saturated pseudo‐optimized model and used AICc to select the top model (Doherty et al., 2012). We only considered model parameters as explanatory when 85% confidence intervals did not include zero (Arnold, 2010).

We developed predictive surfaces using our best model for nest‐site selection to identify important nesting habitats for upland nesting ducks in the boreal forest. We evaluated spatial autocorrelation using bubble plots and model fit using area under the curve (AUC) (Boyce et al., 2002; Fedy et al., 2018; Hirzel et al., 2006).

## 3 RESULTS

We located 136 nests of upland nesting duck species between 2016 and 2018. We located nests of 16 American Wigeon (*Mareca americana*), 54 Blue‐winged Teal (*Spatula discors*), 16 Green‐winged Teal (*Anas crecca*), 12 Lesser Scaup (*Aythya affinis*), 36 Mallard (*Anas platyrhynchos*), and two Northern Shoveler (*Anas clypeata*). We modeled upland nesting ducks as an entire guild to compare to other studies (e.g., Lemelin et al., 2007; Singer et al., 2020), because our sample size was not large enough to explore species‐specific effects at the landscape scale and because we assumed predation effects of oil and gas development would be species independent within upland nesting ducks and therefore, pooling would provide a higher probability of detecting important habitat associations.

**FIGURE 2:**
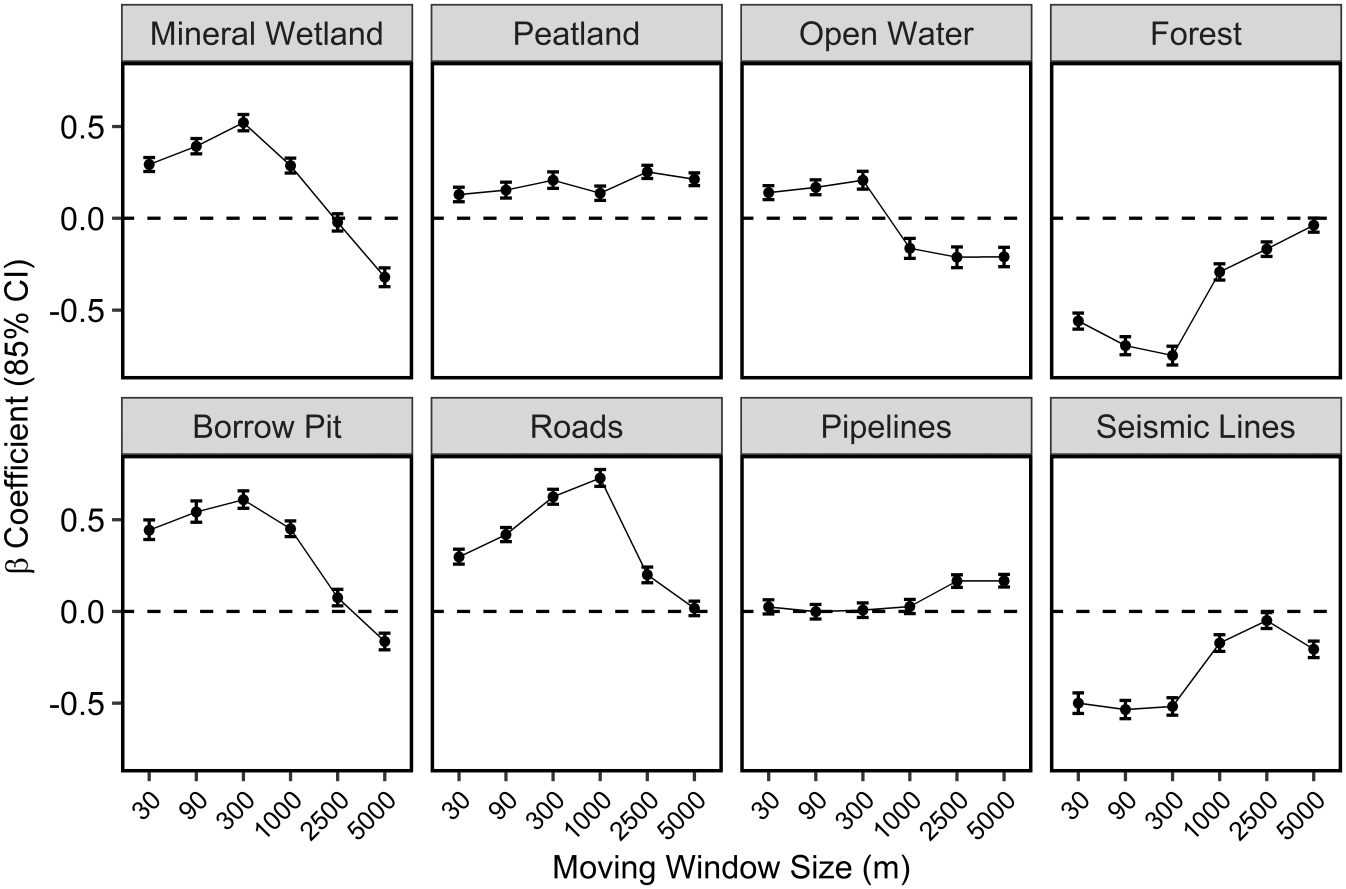
Multiscale functional response curves for land cover and land use variables derived from univariate resource selection functions. Black points represent beta coefficients with 85% confidence intervals. The line joining points is provided for display only and represents trend across scales.

**FIGURE 3:**
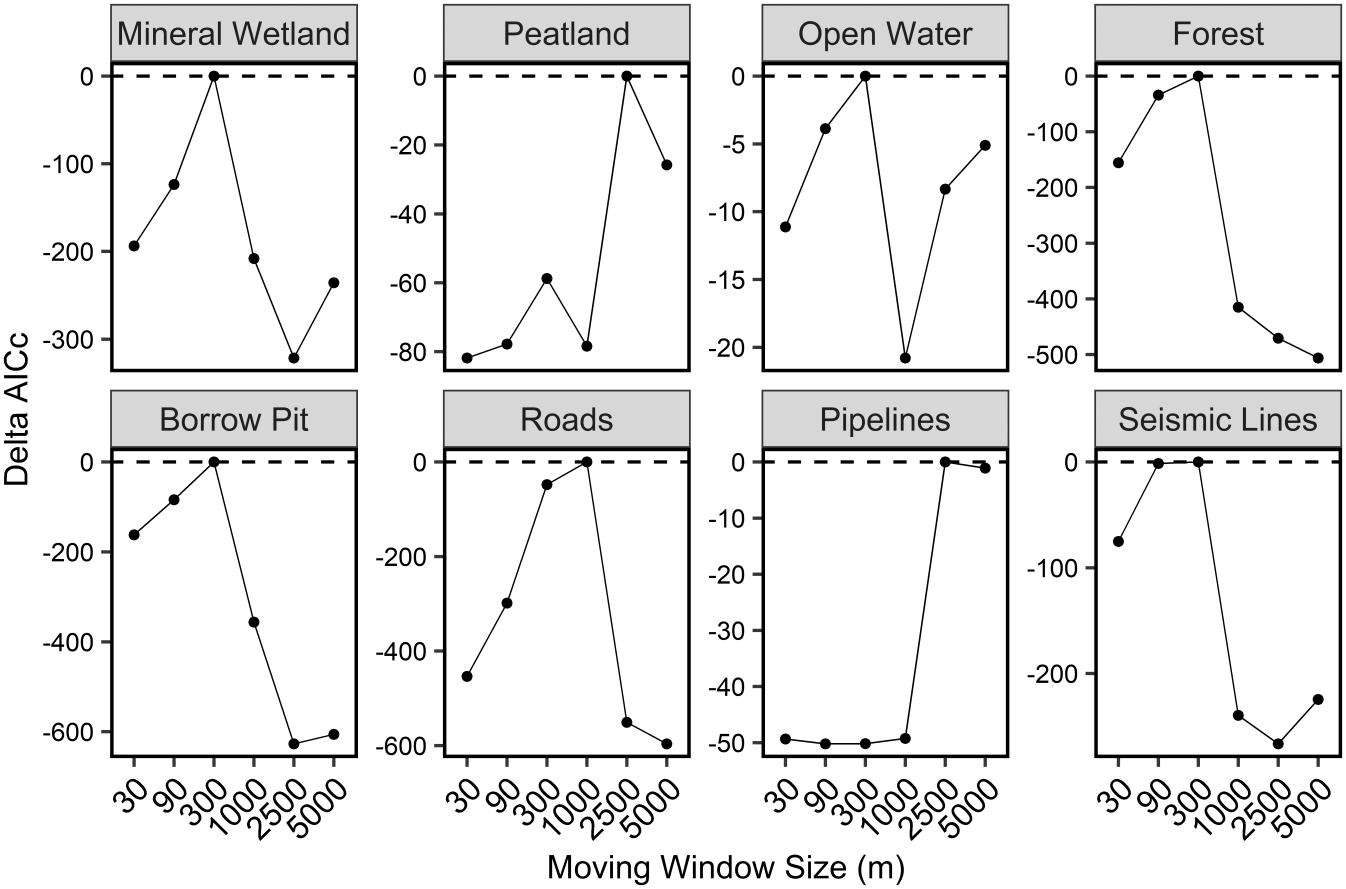
AICc for each scale considered for land use and land cover variables from the univariate resource selection functions. Points at the 0 line indicate the best scale for each individual variable.

We observed ducks selecting different resources at different spatial scales (Figures 2 and 3). Peatland was the only variable to be selected across spatial scales, while seismic lines were consistently avoided. Roads and pipelines were also selected or showed no response across spatial scales, while forests were generally avoided but no response was detected at the largest spatial scale. Mineral wetlands, open water, and borrow pits generally ranged from selection at fine spatial scales to avoidance at coarse spatial scales (Figure 2). The most predictive spatial scale for each covariate, determined by AICc from our univariate models, was: 300 m for mineral wetland, 2500 m for peatland, 300 m for open water, 300 m for forest, 300 m for borrow pit, 1000 m for roads, 2500 m for pipelines, and 300 m for seismic lines (Figure 3). The next most competitive scale for each variable was greater than 2 AICc from the best scale and the direction and magnitude of the effect was similar for closely competing scales (Figure 3).

**FIGURE 4:**
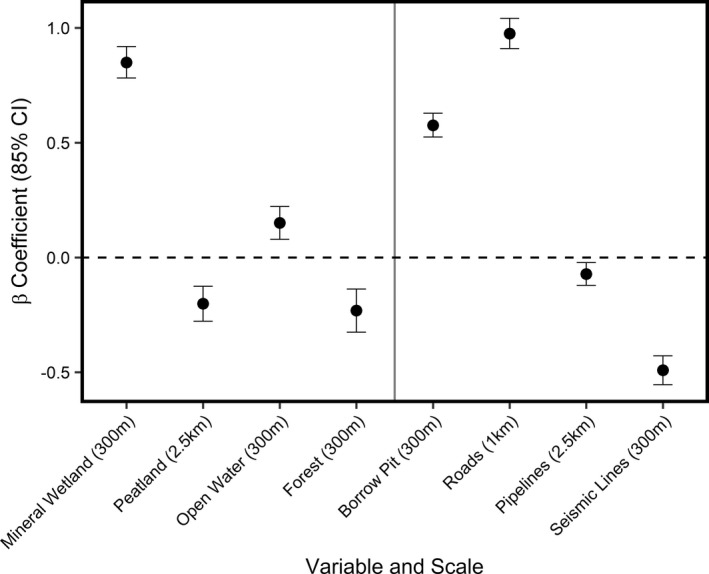
Standardized beta coefficients and 85% confidence intervals from top multiscale resource selection functions for upland nesting ducks in the western boreal forest of Alberta, Canada. The dashed horizontal line indicates no selection, and everything above is selected and below is avoided. The vertical line separates land cover from industrial development variables. The best scale for each variable is provided in parentheses.

**FIGURE 5:**
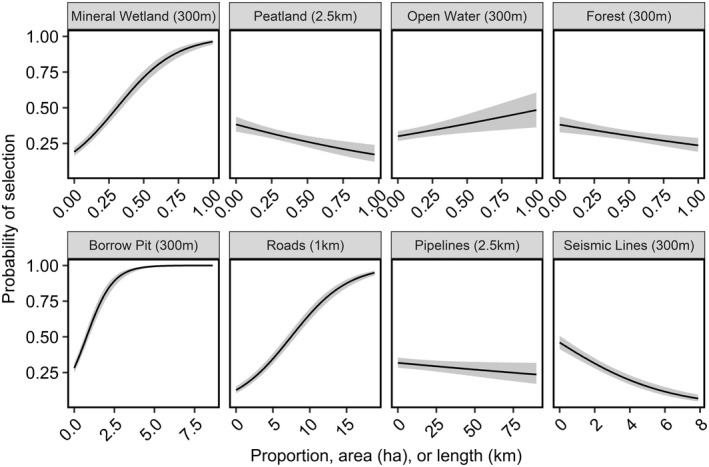
Effect plots for the best multiscale nest‐site selection model for upland nesting ducks in the western boreal forest of Alberta, Canada from 2016 to 2018 with 85% confidence intervals. The best scale for each variable is indicated in parentheses. Units for landcover features are proportions, borrow pits are measured in hectares (ha), and linear features are measured in kilometers (km).

Our best multiscale model for nest‐site selection included the land cover variables mineral wetland, peatland, open water and forest; and industrial development variables borrow pits, roads, pipelines, and seismic lines (Figure 4). Ducks responded to landcover variables at fine (300 m) spatial scales, except for peatlands (2500 m; Figure 4). The steepest response was observed for mineral wetlands, where increasing mineral wetland (*β* = 0.85, 85% CI = 0.78–0.92) land cover resulted in a steep increase in the probability nest‐site selection (Figure 5). The proportion of open water (*β* = 0.15, 85% CI = 0.08–0.22) within 300 m of a nest also increased the probability of selection, while the proportion of forest (*β* = −0.23, 85% CI = −0.33 – −0.14) within 300 m of a nest decreased the probability of selection (Figure 5). Finally, the proportion of peatland (*β* = −0.20, 85% CI = −0.28 – −0.13) within 2500 m of a nest decreased the probability of nest‐site selection.

For industrial development variables, ducks also responded at fine spatial scales (<1000 m), except for pipelines (2500 m). Borrow pits (*β* = 0.58, 85% CI = 0.52–0.63) exhibited the steepest response, where the probability of nest‐site selection sharply increased from 0 to 2.5 ha of borrow pits within 300 m of a nest and was close to 1 when there was greater than 2.5 ha of borrow pits (Figure 5). Nest‐site selection responses also varied with linear feature type. Ducks increased their probability of nest‐site selection with increasing lengths of roads (*β* = 0.98, 85% CI = 0.91–1.04) within 1000 m of a nest site (Figure 5). Conversely, the probability of nest‐site selection decreased with increasing lengths of pipelines (*β* = −0.07, 85% CI = −0.12 – −0.02) within 2500 m of a nest and seismic lines (*β* = −0.49, 85% CI = −0.55 – −0.43) within 300 m of a nest (Figure 5).

**FIGURE 6:**
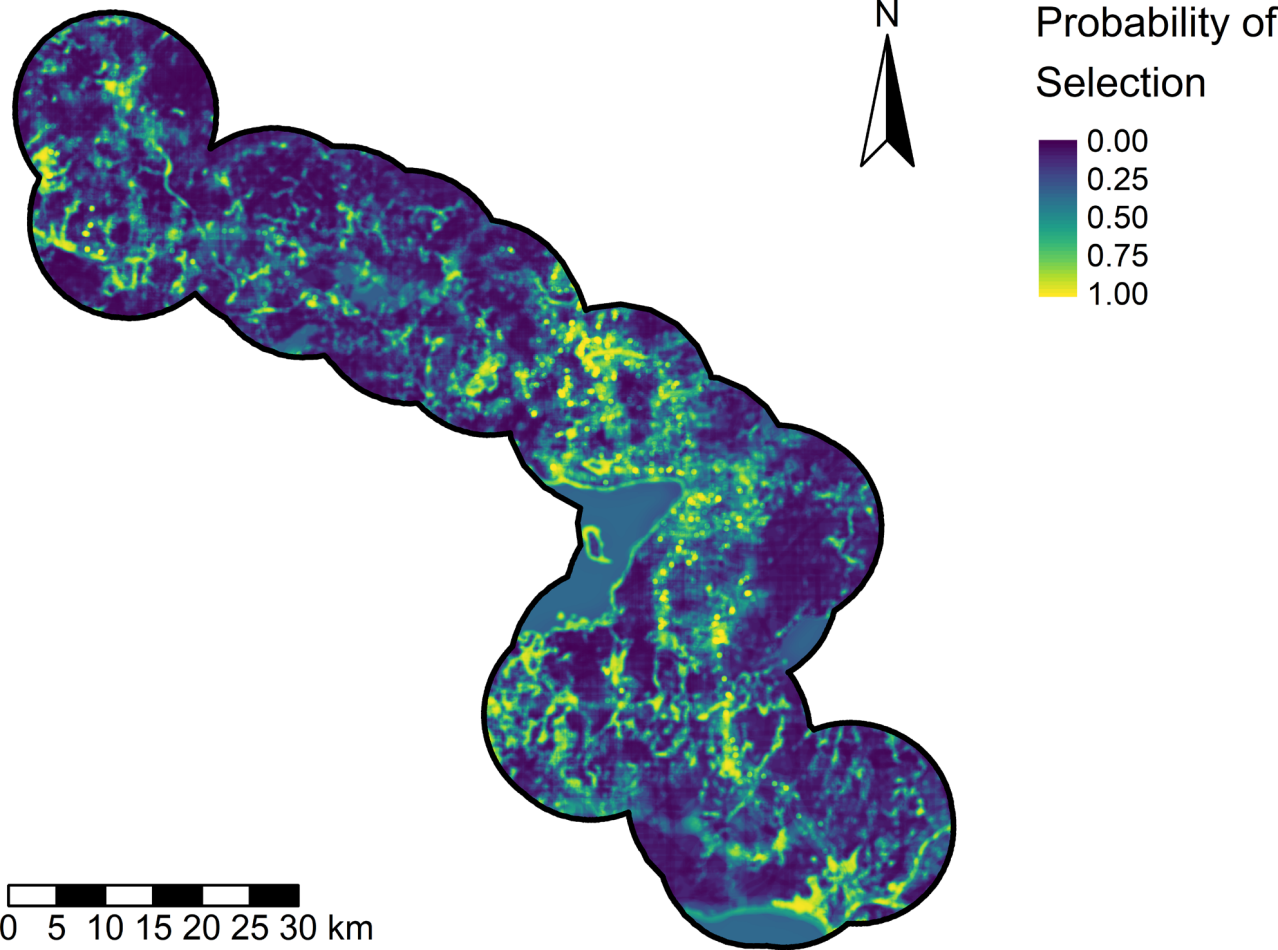
Map of critical nesting habitat for upland nesting ducks in the boreal forest of Alberta, Canada from 2016 to 2018 based on the best multiscale nest‐site selection model.

We spatially predicted (i.e., mapped outputs from) our top model of nest‐site selection for boreal ducks to identify nesting habitat with a high probability of selection (Figure 6). We did not observe any spatial autocorrelation in our predictions and our final top model had an AUC score of 0.89 indicating strong predictive performance.

## 4 DISCUSSION

Our results provide novel insights into how nesting ducks respond to oil and gas development in the boreal forest. Ducks selected nest sites with a greater area of borrow pits and road density but avoided nesting in areas with greater pipeline and seismic line density. Landcover also was an important factor explaining nest‐site selection, highlighting the importance of mineral wetland and open water habitats to ducks in the boreal forest. We provide a tool (i.e., map; Figure 6) that extrapolates our findings to help prioritize conservation of nesting habitats in this region.

Borrow pits are generally rectangular and <3 ha in size (Kuczynski & Paszkowski, 2012) with grassy buffers in an otherwise forested landscape and may provide important microhabitat characteristics selected by nesting ducks (Dyson et al., 2019; Eichholz & Elmberg, 2014). Consistent with our findings, mallard, lesser scaup, bufflehead, ring‐necked duck, green‐winged teal, American wigeon, and American coot (*Fulica americana*) occurred on borrow pits in Alberta's boreal forest and species occurrence was mediated by forest cover (Kuczynski & Paszkowski, 2012). Horned grebe chicks also occurred more on borrow pits with greater amounts of riparian vegetation (i.e., trees and shrubs; Kuczynski et al., 2012), suggesting possible variation in borrow pit quality for reproduction. The quality of borrow pits for breeding ducks, and other waterbirds, warrants further investigation, because they are common on the landscape and are often oligotrophic or mesotrophic with low levels of dissolved oxygen (Stevens et al., 2006), which may represent habitat sinks for broods if forage is limited.

Meanwhile, linear features such as seismic lines may act as predator corridors (Dickie et al., 2017; McKenzie et al., 2012; Slattery et al., 2011) allowing easier access to nesting habitat and an increased probability of nest predation. For example, wolverines, wolves, and black bears use seismic lines for travel (Dickie et al., 2017; Scrafford et al., 2017; Tigner et al., 2014) and black bears were an important nest predator in our study (Dyson et al., 2020). Thus, coarse scale avoidance of pipelines and seismic lines may reflect avoidance during settling to reduce predation risk. Predation risk was proposed as a plausible mechanism to explain declining trends of upland nesting ducks associated with greater pipeline densities in the boreal forest (Singer et al., 2020). Contrary to pipelines and seismic lines, roads may act as refugia from predators if some duck predator species tend to avoid them (Pasitschniak‐Arts et al., 1998; Roy, 2018). Vegetation associated with road margins often consists of grasses and other dense vegetation species (Emery et al., 2005), which are selected by boreal nesting ducks (Dyson et al., 2019), potentially providing preferred microhabitat conditions. Mallard and blue winged‐teal occupied nest sites close to roads and wells more than expected in the Prairies too (Ludlow & Davis, 2018; Pasitschniak‐Arts et al., 1998), which may have been explained these same mechanisms. Further evaluation of how duck predators respond to these linear features would be valuable in understanding this relationship.

Overall, evidence from research investigating the effects of habitat loss and fragmentation on ducks has identified general resilience to disturbance. For example, upland nesting ducks in Quebec's boreal forest showed no change in abundance in association with clear cuts (Lemelin et al., 2007). In North Dakota's Bakken formation, nest survival was driven mostly by grassland cover and there was no effect detected from oil and gas development (Skaggs et al., 2020). While other evidence from North Dakota suggests a small negative effect of oil and gas development on brood abundance; however, the effect was only evident for a small percentage of the population (Kemink et al., 2019) and there was no evidence for pair avoidance during settling (Loesch et al., 2021).

The boreal forest is a peatland dominated landscape interspersed with a mosaic of upland forest habitat, open water, and mineral wetlands. Mineral wetlands form the intermediary transition between open water and peatlands or forest habitats (Smith et al., 2007) and were selected for nesting by ducks in our study. Importantly, ducks are not nesting in mineral wetlands or open water per se; rather they are nesting in locations with a greater composition of these habitats in proximity (i.e., 300 m) to nests. Mineral wetlands likely provide hens with flooded foraging habitat during pre‐nesting and incubation and brood‐rearing habitat post‐hatch (Bloom et al., 2012). Ducklings hatch precocial but flightless and therefore early brood‐rearing habitat close to nests increases survival (Bloom et al., 2012; Dyson et al., 2018). In North Dakota, wetland size and emergent cover were the strongest predictors of brood abundance in association with oil and gas development at the finest spatial scale of investigation (320 m; Kemink et al., 2019). Selection of open water in our study was not consistent across scales, where selection occurred at finer scales (<1000 m) but avoidance occurred at coarser spatial scales (>1000 m). One interpretation is that boreal ducks likely use smaller ponds, or open water areas, associated with nests like ducks elsewhere in North America (Baldassarre, 2014; Batt et al., 1992; Gilmer et al., 1975; Krapu et al., 1997). Following that logic, the avoidance of open water at coarser scales indicates the avoidance of larger bodies of water for nesting (e.g., lakes).

While peatland and forest habitat were avoided, they also had the highest use, suggesting that open water and mineral wetland habitats within a matrix of peatland and forest cover provides nesting habitat for ducks on this landscape. The avoidance of peatland at a coarse spatial scale suggests that large peatland complexes without the interspersion of open water or mineral wetlands provided little nesting value to ducks, which is reflected in our model prediction (i.e., map; Figure 6). At a finer spatial scale, the avoidance of forest cover suggests that ducks generally avoided nesting in the forest or at least closer to forest edges, which may reduce duckling mortality if overland travel for ducklings is riskier further from wetlands (Chouinard Jr & Arnold, 2007; Simpson et al., 2007). In grassland dominated systems, negative effects of trees were not found in association with nest success (Thompson et al., 2012). However, trees have been suggested to provide perches for avian predators, like raptors, causing a tradeoff between nest and hen survival (Devries et al., 2003).

We caution readers to interpret our results with the following caveats. Ducks nest across a forest to open habitat gradient with species such as American wigeon, and green‐winged teal nesting in forests; mallards using both forests and open habitats, and blue‐winged teal using more open habitats (Baldassarre, 2014; Dyson et al., 2019; Keith, 1961). Our sample of nests largely consisted of blue‐winged teal and mallard nests (66%). Therefore, the species composition of our sample may drive the avoidance of forest cover and selection of roads for upland nesting ducks (Ludlow & Davis, 2018; Pasitschniak‐Arts et al., 1998). We also targeted our nest searching efforts on predicted areas of high density of settling ducks, which resulted in generally under sampling available forest landcover further from wetlands where detection is also predicted to be reduced (Petrula, 1994). We suspect that if large numbers of ducks were nesting further into the forest, we would have located more nests further from wetlands than we did given our search efforts and other co‐occurring field work (i.e., telemetry, incidental encounters; Dyson, 2020). Targeted search efforts in forest habitat further from wetlands or expanded use of telemetry would provide important additional information.

Our research adds to the body of literature aimed at understanding changes to ecosystem structure and function driven by industrial development in the boreal forest (Bayne et al., 2016; Fisher & Burton, 2018; Mahon et al., 2019; Shonfield & Bayne, 2017; Tattersall et al., 2020). To benefit ducks, we suggest continued conservation efforts that focus on mineral wetland and open water habitats interspersed within the peatland and forest dominated landscape in areas with low densities of pipelines and seismic lines. Our spatial predictions identify where those places are in the study area, providing a decision‐support tool for managers to improve conservation outcomes by better prioritizing key nesting habitats and aiding identification of threats and appropriate conservation actions. However, we caution against extrapolating our predictions outside our study extent without testing its predictive ability against independent data or explicitly accounting for variation in availability at novel sites. We also recognize that a more comprehensive understanding of industrial effects requires study of demographic implications, and so suggest that investigating consequences of nest‐site selection decisions on nest survival is a critical next step.

## AUTHOR CONTRIBUTIONS

**Matthew Dyson:** Conceptualization (equal); data curation (lead); formal analysis (lead); funding acquisition (equal); investigation (lead); methodology (lead); project administration (supporting); visualization (lead); writing – original draft (lead); writing – review and editing (equal). **Stuart Slattery:** Conceptualization (equal); formal analysis (supporting); funding acquisition (equal); investigation (supporting); project administration (supporting); supervision (supporting); writing – original draft (supporting); writing – review and editing (supporting). **Bradley C Fedy:** Conceptualization (equal); data curation (supporting); formal analysis (supporting); funding acquisition (equal); investigation (supporting); methodology (supporting); project administration (lead); supervision (lead); writing – original draft (supporting); writing – review and editing (equal).

## Data Availability

The data that support the findings of this study are openly available at the University of Waterloo's online data repository at https://doi.org/10.5683/SP3/CEKYXV.
